# Biochar in the Agroecosystem–Climate-Change–Sustainability Nexus

**DOI:** 10.3389/fpls.2017.02051

**Published:** 2017-12-11

**Authors:** Vimala D. Nair, P. K. Ramachandran Nair, Biswanath Dari, Andressa M. Freitas, Nilovna Chatterjee, Felipe M. Pinheiro

**Affiliations:** ^1^Soil and Water Sciences Department, University of Florida, Gainesville, FL, United States; ^2^School of Forest Resources and Conservation, University of Florida, Gainesville, FL, United States

**Keywords:** feedstocks, highly weathered tropical soil, low-input agriculture, manure, nutrient retention, phosphorus availability, plant biomass

## Abstract

Interest in the use of biochar in agriculture has increased exponentially during the past decade. Biochar, when applied to soils is reported to enhance soil carbon sequestration and provide other soil productivity benefits such as reduction of bulk density, enhancement of water-holding capacity and nutrient retention, stabilization of soil organic matter, improvement of microbial activities, and heavy-metal sequestration. Furthermore, biochar application could enhance phosphorus availability in highly weathered tropical soils. Converting the locally available feedstocks and farm wastes to biochar could be important under smallholder farming systems as well, and biochar use may have applications in tree nursery production and specialty-crop management. Thus, biochar can contribute substantially to sustainable agriculture. While these benefits and opportunities look attractive, several problems, and bottlenecks remain to be addressed before widespread production and use of biochar becomes popular. The current state of knowledge is based largely on limited small-scale studies under laboratory and greenhouse conditions. Properties of biochar vary with both the feedstock from which it is produced and the method of production. The availability of feedstock as well as the economic merits, energy needs, and environmental risks—if any—of its large-scale production and use remain to be investigated. Nevertheless, available indications suggest that biochar could play a significant role in facing the challenges posed by climate change and threats to agroecosystem sustainability.

## Introduction

Agroecosystems the world over are under severe stress. Faced with the challenge of feeding the burgeoning population and meeting the ever-growing demands for fiber and other natural products, agricultural and forestry production systems have become highly dependent on chemical products and technological inputs (for example, Mueller et al., 2012). While the resultant production increases have helped eradicate hunger in many parts of the world, the accompanying ecosystem degradation on a massive scale has raised major concerns (Nair P. K. R., 2014). Consequently, farming practices and technologies that can increase and sustain production without ruining the ecosystem were promoted as an approach to addressing these concerns. Thus, numerous terms and rallying themes became prominent in the global land-use arena during the past few decades, such as (in alphabetical order), agroecology, agroforestry, climate-smart agriculture, conservation agriculture, organic agriculture, permaculture, sustainable intensification, and so on. Almost all of them share the objective of minimizing external inputs by building on the efficient use of locally available resources. This has led to focusing attention on some naturally occurring materials as well as products that can be relatively easily assembled from natural resources to substitute or complement the use of synthetic products. Biochar is one such product that has become quite prominent in the recent past. This paper presents a synthesis and evaluation of the current level of knowledge on biochar and its potential role in agroecosystem management in the climate–change–sustainability context.

## Properties of biochar: the current state of knowledge

The International Biochar Initiative (IBI) describes biochar as “*a solid material obtained from the carbonisation of biomass”* (http://www.biochar-international.org/) which occurs when biomass (such as wood, manure, or crop residues) is heated in a closed container with little or no air (Lehmann and Joseph, 2009). Consequent to the realization of the potential role of biochar, there has been a veritable explosion of interest in biochar in the scientific community. Several materials are reported to have been used as biochar feedstock in different parts of the world for improving soil fertility and plant nutrition. A summary of the available scientific reports on biochar, especially those during the past 5 years, focused on its properties and role in plant nutrition and soil management is presented in Table 1.

**Table 1:** Summary of major research results reported on the effect of biochar application on plant nutrition and soil nutrient dynamics.

**Study Location**	**Biochar**		**Soils characteristics**		**Crop and Study method**	**Application rate (Mg ha^−1^); Biochar pH**	**Crop yield/growth resp. over control**		**Nutrient and water retention & availability**		**Reference**
	**Feedstocks**	**(Pyrolysis Temp, ^0^C)**	**Description**	**pH**							
China	Wheat straw	350–550	Calcareous loamy and silty clay loam	NA^¶^	Maize and rice; Field	10, 20, 40 with and without N	Corn: 7-12% yield Rice: 8-14% yield	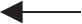	NA^¶^		(1)
	Wheat straw Maize straw	300–600 400	Upland Red soils (~Ultisols)	6.7	Rapeseed and potato; Field	0, 2.5, 5, 10, 20, 30 & 40	Rapeseed: 36% Potato: 54% yield	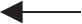	soil water stable aggregate soil organic carbon total N and C:N ratio	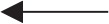	(2)
	Maize straw Wheat straw	400	Sandy loam and Calcic	NA^¶^	Rice and maize; Field	2.4	Rice & maize: 6% yield		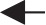 NA^¶^		(2)
	Wheat straw	350–550	Hydroagric Stagnic Anthrosol	NA^¶^	Rice; Field	40	18.3% grain yield		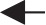 NA^¶^		(3)
	Pig manure compost, peanut husk & biosolids	350–450	Entic Hydroagric Anthrosol	NA^¶^	Rice; Field	0.45	13.5%, 28.1% & 31.4% grain yield		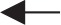 NA^¶^		(4)
	Rice straw	NA^¶^	Gleyi–Stagnic Anthrosol	NA^¶^	Rice-wheat; Pot	4.5 & 9	14.8 & 21.3% grain yield		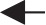		(5)
	Giant reed grass (*Arundo donax*)	300–600	Tropical sandy; 29.2% sand, 13.6% clay	6.02	Maize; Greenhouse column	0, 1, 2, 5% (w/w)	Growth 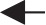		Reduce in ${\text{NH}}_{4}^{+}$-N, Increase in WHC, improve in N bioavailability		(6)
Japan	Chicken manure	402–528	Sandy: 47.5% sand, 11.7% clay	7.0	*Brassica rapa;* Field	10 (pH = 10.5)	90% growth 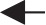		25% mineralization of the total N.		(7)
	Wood-based (Japanese cedar and cypress)	300	Sand-dune soils	6.9	Rice; Field	0, 20 & 40 (pH = 9.8)	Crop yield 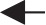		20–30% to 50–60% increase in available water content		(8)
	Rice husk	350–400	Haplic Andosols	NA^¶^	Rice; Pot	0.02, 0.2 & 2 kg m^−2^	14% straw yield 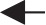		NA^¶^		(9)
USA New York	Maize Stover	600	Kendaia silt loam and Lima loam.	7.36	Maize; Field	0, 1, 3, 12, and 30; + 108 kg N ha^−1^ (pH = 10.0)	No effects on yield		No improvement in crop N use efficiency; N uptake did not change; increased N retention		(10)
Florida	Peanut hull & Brazilian pepperwood	600	Sandy: sand: 94%, clay: 3.0%	5.9	Laboratory column	0.1 g char L^−1^ aqueous solution	Crop yield 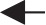		Decrease in nitrate (34%), ammonium (35%) & phosphate (21%) leaching		(11)
Idaho	Hardwood biochar & dairy manure co-application	500	Calcareous; Portnuef soil	8.2	Lab incubation	0%, 1%, 2%, 10% by wt (pH = 6.8)	NA^¶^		Improve in soil water content; increase in soil NO_3_-N		(12)
Spain	Bamboo wood, Dairy manure, & mixed wood chip	NA^¶^	Sandy to silty clay loam	6.5	Lab incubation	2% (w/w; dry weight)	NA^¶^		Consistent decrease in N_2_O emissions by 10–90%		(13)
	Olive-tree prunings	450	Vertisol: 22% sand, 51% clay	8.2	Wheat; Field	2% by weight (pH = 6.6)	Crop yield 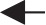		Increase in available N, P and C		(14)
Germany	Peanut hull	NA^¶^	Sandy	6.0	Quinua; Greenhouse	100 and 200 char (pH = 8.1)	Crop yield		Increase in leaf N; decrease in greenhouse gas emissions; increase in WHC		(15)
	Maize biochar used as hydro-biochar	600	Loamy sand	6.2	Wheat; Pot	0, 4, 12 (pH = 7.7)	Crop yield 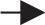		No effect on N and Ca contents; decrease in plant tissue N		(16)
Denmark	Straw	730	Coarse sandy	6.5	Barley; pot	0, 8, 16, 32, 64 + (208 N+30 P) fertilizer	Yield: 6.0, 22, −12, −28, 10% 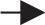		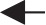 NUE was not prominent		(17)
Australia	Willow wood	550	Tropical Ferralsol	acidic	Maize; Field	0, 10, 25 + compost (co-composting)	10–29% yield		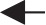 Increase in soil N, P, OC & water content		(18)
Bangladesh	Sawdust	300–350	“Alkaline”	8.0	Soybean; Pot	20 (pH = 5.21)	54% yield 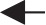		Increase in available P		(19)
Finland	Spruce chips (Picea abies)	550–600	Boreal loamy sand 83% sand, 2% clay	4.65	Wheat; Field	0, 5, 10, 20, 30 + inorganic fertilizers	No effects on yield		Increase in soluble K & SOC; no effects on other soil nutrients (N, P); increase in plant-available water content		(20)
Indonesia	Bark of *Acacia mangium*	260–360	“Acidic” soil		Maize; Field	37	12% yield 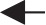		NA^¶^		(21)
Philippines	Rice husk (Chimney charring process)	NA^¶^	Anthraquic gleysols Humic nitisols	6.55 4.3	Rice; Field	4.13 kg m^−2^	Both 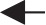 (16-35%) & 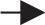 in yield		NA^¶^		(22)

*BC, biochar; C, Carbon; Ca, calcium; CUE, cation exchange capacity; K, potassium; N, nitrogen; NUE, nitrogen use efficiency; OC, organic carbon; P, phosphorus; WHC, water-holding capacity*.

¶ *NA, not available. The up arrows and down arrows represent, respectively, the increasing and decreasing responses of the parameters by biochar application*.

*References*:

(1) *Zhang et al., 2016*;

(2) *Liu et al., 2014*;

(3) *Bian et al., 2014*;

(4) *Qian et al., 2014*;

(5) *Zhao et al., 2014*;

(6) *Zheng et al., 2013*;

(7) *Ishimori et al., 2017*;

(8) *Kameyama et al., 2017*;

(9) *Koyama and Hayashi, 2017*;

(10) *Guerena et al., 2013*;

(11) *Yao et al., 2012*;

(12) *Ippolito et al., 2016*;

(13) *Cayuela et al., 2013*;

(14) *Olmo et al., 2014*;

(15) *Kammann et al., 2011*;

(16) *Reibe et al., 2015*;

(17) *Bruun et al., 2012*;

(18) *Agegnehu et al., 2016*;

(19) *Mete et al., 2015*;

(20) *Tammeorg et al., 2014*;

(21) *Yamato et al., 2006*;

(22) *Haefele et al., 2011*.

### Biochar as a source of plant nutrients

Recent research has showed that elemental composition of a feedstock is not an indication of plant-nutrient availability in the biochar made out of that feedstock. Freitas et al. (2016) found that available P (Mehlich 3) in biochar made from different feedstocks was not at all proportional to the total P concentration of the feedstocks. X-ray diffraction showed that poultry litter biochar contained the mineral whitlockite (a sparingly soluble Ca-P or Ca-Mg-P form), which might be used as a slow-release P fertilizer (Dari et al., 2016). Furthermore, Mehlich 3-extractable *K*-values in biochar from different feedstocks were also not proportional to the concentration of the nutrients in the “parent” feedstock. Based on these, Freitas et al. (2016) suggested that some nutrient contents of animal-based biochar (e.g., K) would not necessarily be higher than those of plant-based biochars.

The existence of such variability in biochar properties has been well-established (Ippolito et al., 2015), but information on the reasons for such differences is scanty. Pyrolysis is conducted at varying temperatures (Table 1), and the temperature is reported to have effect on the quality of biochar produced; a definitive relationship between the two, however, has not been established. Other processing differences could lead to different biochar properties. Thus, it could well be that biochars prepared from the same feedstock could have different characteristics depending on pyrolysis temperature and other conditions.

Biochar + compost mixtures are becoming popular for improving soil fertility and plant growth (Schulz and Glaser, 2012; Prost et al., 2013), especially when biochar is mixed with biomass before composting. A recent review by Godlewska et al. (2017) has pointed out that the effect of biochar on composting depends on biochar and feedstock properties. Some studies indicate the formation of oxygen-containing functional groups during composting, which leads to increase in nutrient retention (Schulz et al., 2014). This practice allows a higher nutrient retention in the biomass, adding to the value of the final product. As concluded by Wu et al. (2017) in another recent review, biochar and composting could alter the physico-chemical properties of both materials. The combination of biochar with compost seems to be a promising source of amendment and an interesting alternative to inorganic fertilizer.

## Relationship between biochar and soil properties

### Soil nutrient retention

Nutrient retention/loss risk during biochar application depends not only on the nutrient release potential of the biochar, but also the nutrient retention properties of the soil. Dari et al. (2016) showed that P retention in non-calcareous soils is a property of the soil, independent of the nature of the feedstock. Therefore, the biochar from the same source added at a given rate to different soils could have different effects based on the respective soil properties. As in the case of inorganic P additions, any P released by a given biochar will be retained by the soil as long as the threshold P saturation ratio of the non-calcareous soil is not exceeded (Nair V. D., 2014). For example, when the same amount of biochar is added to a sandy soil and a more clayey soil, the sandy soil will begin to release P faster than the clayey soil. The temperature at which biochar is produced may not have any effect on P release property of the biochar-amended soil (Nair et al., 2016); therefore biochar produced using sophisticated techniques or in simple kilns would likely behave similarly on a given soil type.

### Soil aggregate formation and stabilization of soil organic matter

The influence of biochar on soil aggregates and physical stabilization of soil organic matter (SOM) in aggregates has been relatively less studied. Wang et al. (2017) demonstrated that, on addition of biochar, soil aggregation markedly differed between two contrasting soils: while biochar amendment dramatically improved aggregate stability in a fine-textured soil, it had no significant impact on a coarse-textured soil. Biochar also increased C storage in macroaggregates of the fine-textured soil and thereby enhanced the physical protection of SOM in the soil by increasing the proportion of C stored within macroaggregates. On the other hand, Fungo et al. (2017) did not find any effect of biochar addition on soil aggregation in a 2-year study on a tropical Ultisol. These studies suggest the effect of biochar addition on soil aggregation and organic matter stabilization is variable depending on the soil type.

### Soil physical properties

Several studies have reported that biochar addition to soils decreases soil bulk density (BD) and increases water-holding capacity (WHC). Increase in WHC following biochar addition is attributed to high surface area and porosity of biochar (Novak et al., 2009; Kinney et al., 2012; Laghari et al., 2016), which contributes to greater water use efficiency and thus plant productivity. Increase in WHC by biochar additions could be particularly pronounced in sandy soils, where the low surface area of their particles and abundance of macro-pores limit the capacity for holding water. Based on studies using pine-sawdust biochar produced under different temperatures, Laghari et al. (2016) suggested that WHC of desert soils could be improved leading to better plant growth through biochar addition.

### Soil microbial properties

Thies et al. (2015) reviewed the studies on the influence of biochar on soil microbial properties including microbial biomass, enzyme activities, nitrogen mineralization rates, soil respiration, ratio of bacteria to fungi, and soil- borne diseases. Given the variations among different types of biochar, the interaction effects of biochar with various soils and plants under different climatic conditions can be enormously variable. Consequently, there could be corresponding impacts on plant growth and productivity as well as emission of greenhouse gases.

## Biochar in soil carbon sequestration and climate-change mitigation

Based on the management practice of the ancient civilizations, the idea of sequestering carbon via biochar addition to soil has been of interest to scientists as a means of mitigating global warming through soil C sequestration. So much so, biochar application to agricultural soils is now considered as a soil-based greenhouse mitigation strategy for sustainable environmental management (Paustian et al., 2016). Management practices that could potentially increase C sequestration in biomass and in the soil by using biochar as a nutrient source also have received some research attention. Following an evaluation of the characteristics of 76 biochars from 40 studies, Brassard et al. (2016) reported that biochars with lower N content (C/N ratio >30) were found to be more suitable for mitigation of N_2_O emissions from soil, and those produced at higher pyrolysis temperature might have high C sequestration potential.

One of the important attributes of biochar is that carbon in biochar resists decomposition. Lehmann et al. (2006) reported that biochar “can hold carbon in soils for hundreds to thousands of years” as evidenced by the *Terra Preta* soils of the Amazonian region in Northern Brazil (Glaser et al., 2001). A meta-analysis of decomposition and priming effects on biochar stability in soil (Wang et al., 2016) suggested that only a small percentage of biochar C (3%) is bioavailable and that the remaining contributed to long-term stability in soil. The analysis was based on 128 observations of biochar-derived CO_2_ from 24 studies with ^13^C and radioactive ^14^C isotopes. However, a systematic review by Gurwick et al. (2013) concluded that: “there are not enough data to draw conclusions about how biochar production and application affect whole-system GHS (greenhouse gas) budgets.”

Increasing biomass production, whether for increasing food production, energy generation or for reclaiming degraded land, will remove atmospheric CO_2_ and could thus be a mitigating strategy for reducing global warming. Moreover, conversion of agriculture and forestry byproducts into biochar could reduce CO_2_ and methane emissions from feedstocks during the natural decomposition or burning of the waste material (http://www.biochar-international.org/biochar/carbon). Overall, it seems reasonable to conclude that biochar's effect on climate change mitigation cannot be established as a cause—effect relationship; but there could be advantages in the longer term.

## Biochar and sustainable agriculture

Sustainability is another “all-encompassing” and difficult-to-measure issue, such that the specific role of biochar in the sustainability paradigm is rather nebulous, just as for climate-change mitigation. A meta-analysis on the effect of biochar and plant productivity/nutrient cycling (Biederman and Harpole, 2012) indicated that there was increased aboveground productivity, crop yield, soil microbial biomass, rhizobial nodulation, and plant K tissue concentration. The authors also indicated that pH, N, P, K and total C in the soil increased compared to control conditions. Jeffery et al. (2013) commented that while meta-analyses are powerful tools for obtaining insights from published literature, they rely heavily on input data, a view the authors of this paper share. Additionally, almost all the issues discussed under effect of biochar on soil properties and many more have relevance to the sustainability issue.

## Limitations of biochar use

Based on available data, Mukherjee and Lal (2014) identified several negative aspects of biochar application to soil. These included leaching losses of C and N, contaminant mobility, and several unfavorable physical changes and changes to soil biota. The authors also identified some negative impacts on agronomic yields, and pointed out that effects of biochar applications on gaseous emissions were contradictory. As Table 1 that summarizes some of the relatively recent literature on the effect of biochar application on plant nutrition and soil nutrient dynamics shows, the majority of the studies reported positive responses, while a few indicated negative ones. It is also likely that some authors may be reluctant to report negative results.

## Opportunities for biochar use

### Land-application of biochar

Besides greenhouse and laboratory experiments, some field studies have been reported on agricultural use of biochar as a nutrient source and soil amendment (Table 1). However, as concluded in a recent review by Agegnehu et al. (2017), a substantial and scientifically rigorous body of knowledge based on large-scale field validation of the purported merits of biochar has not yet been generated. Based on a meta-analysis of the effects of biochar-application on crop yields, Jeffery et al. (2015) concluded that: “while biochar has been shown to have promise for increasing crop productivity, we do not have a mechanistic understanding of the interactions behind observed yield increases to provide universally applicable guidance.” In another meta-analysis, Jeffery et al. (2017) reported that the extent and cause of the assumed yield benefit of biochar use was controversial, and that the yield benefits were from nutrient-poor, acidic, tropical soils when high-nutrient biochar inputs were added. The authors also cautioned that the lack of uniformity in the available literature on biochar effects on crop yield could impact the statistical rigor of such meta-analyses.

### Low input agriculture

The opportunities for using biochar in the low-input agricultural systems that are predominant in developing countries are also worth serious consideration. The smallholder family farms are the mainstay of agriculture in the tropical and subtropical regions. According to FAO statistics, there are 562 M of the so-called small farms out of the total 609 M farms globally. The average size of these farms varies widely among societies and regions, and collectively they account for only 1,260 M ha or roughly 25% of the total agricultural area (http://faostat3.fao.org/faostat-gateway/go/to/home/E). Yet, an estimated 2.6 billion people produce more than 70% of the world's food on these family farms. These smallholder farmers depend heavily on indigenous and locally available materials such as farmyard manure, green manure, and crop residues as soil-fertility resources with only limited use of purchased chemical fertilizers. At the same time, large quantities of agricultural byproducts such as cereal straw and husk, bagasse, and tree limbs that are generated from those multi-species smallholder land-use systems are currently ignored and denigrated as “agricultural waste.”

Highly weathered tropical soils are inherently poor in soil fertility because of numerous physical, chemical, and microbiological constraints that limit agricultural production. Available results on the beneficial effect of biochar application to soils in terms of better nutrient relations (e.g., improving P availability, and reducing nutrient leaching), improvement of soil aeration and water-holding capacity, and enhanced microbiological activities (e.g., symbiotic N_2_ fixation and mycorrhizal associations) suggest the promising role of biochar under these tropical farming systems. Developing appropriate technologies for converting these “waste” products into biochar could go a long way in enhancing crop yields and maintaining soil health. That will be a “win-win” situation in terms of yield increases and waste disposal for smallholder farmers of developing nations.

The multispecies combinations consisting of intimate association of plants of various types and forms including herbs, shrubs, vines, and trees, all in the same production unit, as in agroforestry systems that are common in many parts of the world might be another niche opportunity for biochar technology adaptation. Farm “wastes” of various types become available in relatively large quantities in land-use systems involving frequently harvested tree crops such as palms, coffee (*Coffea* spp), cacao (*Theobroma cacao*), and a variety of other crops. Promising reports are available on the successful conversion of these byproducts and wastes such as coconut (*Cocos nucifera*) husks, shells and sheaths, outer covering of cacao pods, and a variety of other materials to biochar. Obviously, such operations are of limited scale and applicability, but are important, especially in the production of specialty crops and horticultural industry. It will be a worthwhile effort to undertake market surveys and feasibility assessments of such promising endeavors. Indeed, the whole area of socioeconomics of biochar use in low-input agricultural systems deserves serious attention.

### Forestry and specialty crops

The potential for biochar applications in forestry, horticulture, and specialty crops is another area that has not been explored seriously. Production of healthy and vigorous seedlings/saplings is of utmost importance in forestry, landscaping and environmental horticulture, fruit trees, commercial plantings of rubber (*Hevea brasiliensis*), oil palm (*Elaeis guaneensis*), tree spices, and such other perennial specialty crops. Given the reported benefits of biochar and the relatively small quantity of biochar that is needed for application to nursery beds and pots (as opposed to field application for crops), both commercial and small-scale nurseries and individual owners of any size of land holdings could be benefitted by biochar use. Spot application of biochar in planting pits of trees is yet another, relatively unexplored opportunity. For example, establishing nitrogen-fixing trees (NFT) in agroforestry systems in acid soils is a challenge because most NFTs as well as the symbiotic nitrogen-fixing bacteria (*Rhizobium* spp.) prefer pH above 5.5 and many humid tropical soils have pH lower than that. Spot application of lime in tree-planting pits is a commonly adopted practice in such situations. Given its reported soil-amendment-, pH-moderating-, and other beneficial effects, biochar could possibly be applied to such planting pits alone or in combination with lime. The high water-holding capacity of biochar could be particularly advantageous in arid and semiarid regions.

## Conclusions

**Figure 1:**
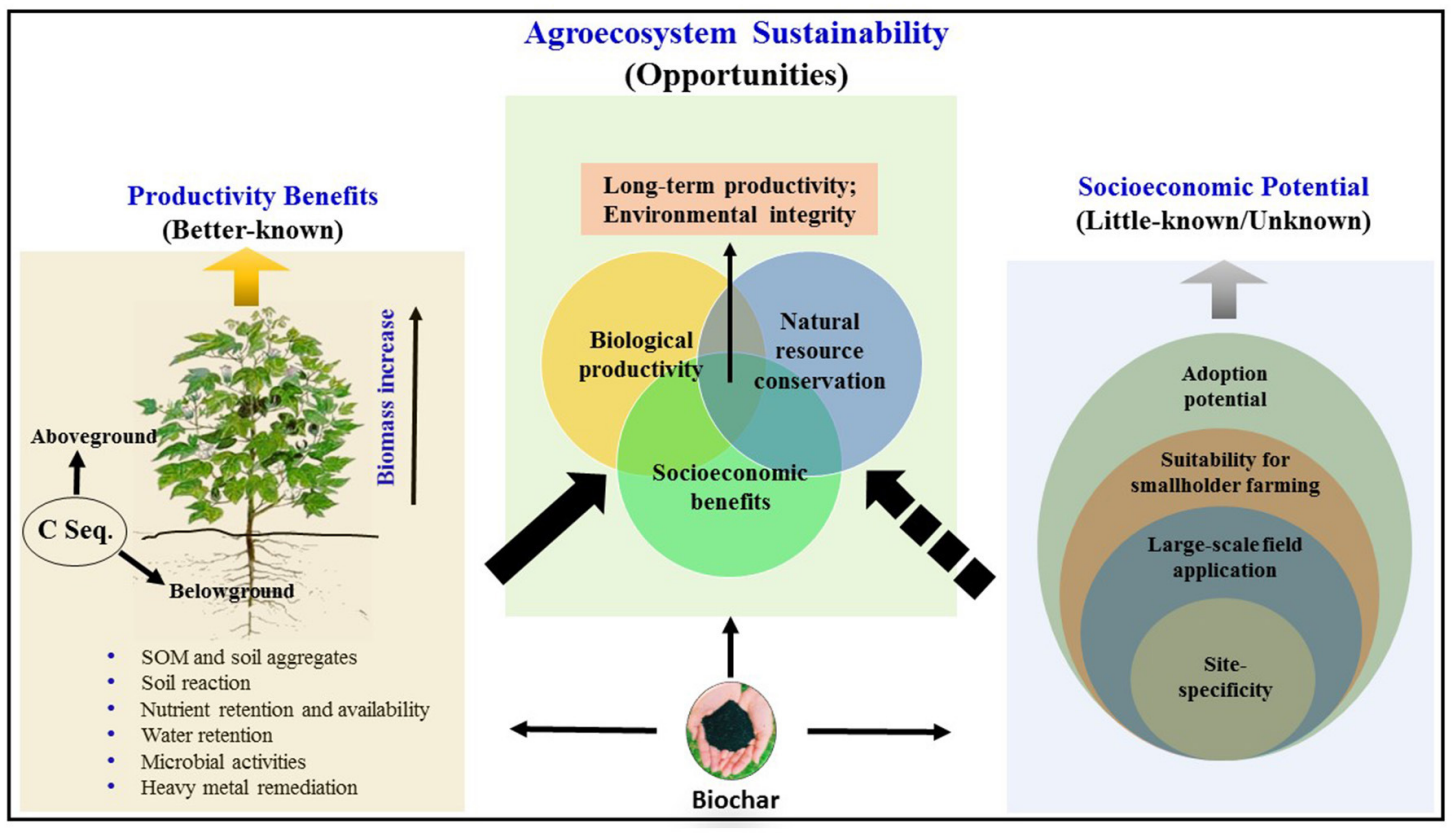
A schematic presentation of the role and potential of biochar in the agroecosystem–climate-change–sustainability nexus. Integration of relatively better-known productivity benefits with the yet-to-be-found solutions to little-known and unknown factors is conceptualized. C = carbon; C seq. = carbon sequestration; SOM = soil organic matter.

Available evidence and indications strongly justify continued research and development efforts in understanding more about the benefits and potentials as well as limitations of biochar and expanding its use in land management. The beneficial role of biochar application on the broader issues of climate-change mitigation and sustainable agriculture can reasonably be assumed based on the available body of knowledge, but it is abysmally weak—almost non-existent—on socioeconomic issues (the “other hand” of sustainability). In order to accomplish the goal of agroecosystem sustainability, it is essential that the two sectors are strengthened and are then properly integrated as presented schematically in Figure 1. Rather than presenting a long “wish list” of “things to do,” suffice it to say emphatically that while biochar use is not a panacea for solving all the problems of land management, it certainly is an aspect that deserves serious attention in agroecosystem management in the future.

## Author contributions

VN: Conceptualized the scope and framework, drafted some sections, and coordinated the efforts. PN: Put together the first draft together with VN and conceptualized Figure 1. BD: Developed Figure 1, Table 1, and assisted in information gathering and discussion. AF, NC, and FP: Collected and collated literature, helped with preparation of Table 1 and section drafts, and participated in discussion. AF also put the reference list together. All authors have read and approved the submitted manuscript.

### Conflict of interest statement

The authors declare that the research was conducted in the absence of any commercial or financial relationships that could be construed as a potential conflict of interest.
